# A Silicon-based Coral-like Nanostructured Microfluidics to Isolate Rare Cells in Human Circulation: Validation by SK-BR-3 Cancer Cell Line and Its Utility in Circulating Fetal Nucleated Red Blood Cells

**DOI:** 10.3390/mi10020132

**Published:** 2019-02-17

**Authors:** Gwo-Chin Ma, Wen-Hsiang Lin, Chung-Er Huang, Ting-Yu Chang, Jia-Yun Liu, Ya-Jun Yang, Mei-Hui Lee, Wan-Ju Wu, Yun-Shiang Chang, Ming Chen

**Affiliations:** 1 Department of Genomic Medicine and Center for Medical Genetics, Changhua Christian Hospital; and Department of Genomic Science and Technology, Changhua Christian Hospital Healthcare System, Changhua 50046, Taiwan; 128729@cch.org.tw (G.-C.M.); 397620cch@gmail.com (W.-H.L.); taiwanbird@gmail.com (T.-Y.C.); 182011@cch.org.tw (J.-Y.L.); 157097@cch.org.tw (Y.-J.Y.); 29561@cch.org.tw (M.-H.L.); 2 Department of Medical Laboratory Science and Biotechnology, Central Taiwan University of Science and Technology, Taichung 40601, Taiwan; 3 International College of Semiconductor Technology, National Chiao Tung University, Hsinchu 30010, Taiwan; cehuang5858@gmail.com; 4 Cytoaurora Biotechnologies, Inc. Hsinchu Science Park, Hsinchu 30016, Taiwan; 5 Department of Obstetrics and Gynecology, Changhua Christian Hospital, Changhua 50006, Taiwan; crystalwu835@gmail.com; 6 Department of Molecular Biotechnology, Da-Yeh University, Changhua 51591, Taiwan; 7 Department of Obstetrics and Gynecology, College of Medicine, National Taiwan University, Taipei 10041, Taiwan; 8 Department of Medical Genetics, National Taiwan University Hospital, Taipei 10041, Taiwan; 9 Department of Life Science, Tunghai University, Taichung 40704, Taiwan

**Keywords:** cbNIPD, fnRBC, capture efficiency, microfluidics, nanostructure

## Abstract

Circulating fetal cells (CFCs) in maternal blood are rare but have a strong potential to be the target for noninvasive prenatal diagnosis (NIPD). “Cell Reveal^TM^ system” is a silicon-based microfluidic platform capable to capture rare cell populations in human circulation. The platform is recently optimized to enhance the capture efficiency and system automation. In this study, spiking tests of SK-BR-3 breast cancer cells were used for the evaluation of capture efficiency. Then, peripheral bloods from 14 pregnant women whose fetuses have evidenced non-maternal genomic markers (e.g., de novo pathogenic copy number changes) were tested for the capture of circulating fetal nucleated red blood cells (fnRBCs). Captured cells were subjected to fluorescent in situ hybridization (FISH) on chip or recovered by an automated cell picker for molecular genetic analyses. The capture rate for the spiking tests is estimated as 88.1%. For the prenatal study, 2–71 fnRBCs were successfully captured from 2 mL of maternal blood in all pregnant women. The captured fnRBCs were verified to be from fetal origin. Our results demonstrated that the Cell Reveal^TM^ system has a high capture efficiency and can be used for fnRBC capture that is feasible for the genetic diagnosis of fetuses without invasive procedures.

## 1. Introduction

Since the first report of circulating fetal cells (CFCs) in maternal blood in 1959 [1], CFCs have been expected as the potential target of noninvasive prenatal diagnosis (NIPD). However, the isolation of CFCs for genetic analysis is always a challenge because of the scarcity of the cells in maternal circulation (1/10,000–1,000,000) [2]. Recently, by advances in knowledge about CFCs and in technology at single-cell genetic analyses, cell-based NIPD (cbNIPD) have again been in focus [3]. In contrast to the popular noninvasive prenatal testing (NIPT) based on cell-free fetal DNA (cffDNA) [4,5,6,7,8,9,10,11,12,13], which mainly reflects the genetic complement of placental trophoblasts and cannot recognize the condition of fetoplacental mosaicism (a situation where there is a discrepancy between the genomic makeup of the fetus and placenta) [14,15], cell-based technology had been reported to be able to capture not only trophoblasts but also fetal nucleated red blood cells (fnRBC, which can truly reflect the fetal genome). Nevertheless, most previous reports regarding cbNIPD focused on capturing trophoblasts from placenta that prohibited a definite diagnosis of fetuses and thus were not superior to cffDNA testing [16,17,18]. One major criticism of the previous studies is that very few fetal specific antigens are available since nucleated red blood cells (nRBCs) in maternal circulation can be of both maternal and fetal origin [19,20]. It is mandatory to verify that the captured nRBCs are indeed of fetal origin. 

Only a few research groups published study results on capturing fnRBCs [19,21,22,23,24]. In our previous report, we have verified our captured circulating nRBCs were indeed of fetal origin using whole genome amplification (WGA) followed by subsequent short tandem repeat (STR) analyses, with a limited sample size (*n* = 5) [19]. There are two directions to solve this hurdle: one is to explore more fetal specific antigens to undoubtedly identify fnRBCs [25,26,27] and the other is to optimize the efficiency of the cell capture platform used. In this study, we adopted the latter strategy to overcome this difficulty by demonstrating that at least a significant proportion of the captured nRBCs are fetal origin, in contrast to most previous reports that showed a rarity of fnRBCs (one in 30 mL maternal blood) by their capturing methodologies [3,28,29].

Rare cell populations in human circulation (i.e., CFCs and circulating tumor cells (CTCs)) can be isolated by different methodologies [30,31,32,33,34,35,36], including (1) immunoaffinity-based positive/negative enrichment; (2) biophysical-based selections by density gradient, size, electrical signature, or acoustophoretic mobility; (3) direct image modalities either by improving the efficiency of imaging or by replacing the enrichment through high-speed fluorescent imaging [37]; and (4) functional assays based on the bioactivity of cells such as protein secretion or cell adhesion [33]. Our platform (named Cell Reveal^TM^ system) is classified as an immunoaffinity-based positive enrichment system coupled with a proprietary direct imaging modality which can accurately map the coordinates of the cells captured, followed by the subsequent recovery of the captured cells by an automated cell picker upgraded from a manual micropipetting system [19]. The microfluidics we used was named as “Coral Chip”, an upgraded version of the PicoBioChip [19], for its coral-like nanostructure clearly visible under the scanning electronic microscope (SEM).

In this study, we evaluate the capture efficiency of the Cell Reveal^TM^ system by spiking tests of SK-BR-3 breast cancer cells. Both array comparative genomic hybridization (aCGH) and next generation sequencing (NGS) were used to elucidate the characteristic molecular signatures of such cancer cells. Then, we validate the use of the platform for a series of prenatal cases in which at least one undisputable non-maternal genomic marker is present in the fetuses, for example, in those women who carried male fetus (Y chromosome will be the non-maternal marker) and in those women with de novo genomic imbalances such as trisomies or chromosome copy number changes. Genetic analyses, including fluorescence in situ hybridization (FISH), aCGH, and STR analyses, were directly performed for the captured cells, which confirm the captured nRBC are indeed from fetuses (i.e., fnRBCs). Our results demonstrated that by capturing fnRBCs and using the subsequent well-established comprehensive genomic approaches, a true NIPD with resolutions similar to the invasive sampling is closer to reality.

## 2. Materials and Methods 

### 2.1. Materials

Two cell lines were used to create artificial cell mixtures in the cell spiking test: (1) SK-BR-3 (human breast cancer cells, HTB-30, ATCC, Manassas, VA, USA), which expresses the cell markers of epithelial cell adhesion molecule (EpCAM) and cytokeratin (CK) and lacks the leukocyte common antigen (CD45). SK-BR-3 cancer cells were maintained in McCoy’s 5A medium (BioConcept, Allschwil, Switzerland), supplemented with 10% fetal bovine serum (FBS) and 100 units/mL antibiotic-antimycotic (Gibco, Grand island, NY, USA). The other cell line was (2) Jurkat (immortalized human T lymphocyte cells), which expresses the cell marker of CD45 and lacks EpCAM and CK. Jurkat cells were maintained in an RPMI-1640 medium (BioConcept, Allschwil, Switzerland), supplemented with 10% FBS and 100 units/mL antibiotic-antimycotic (Gibco, Grand island, NY, USA). Prior to be mixed, both cell lines were incubated with anti-EpCAM antibody at 37 °C for 45 min and then spun at 300× g for 10 min to collect the cell pellets. The cell mixture was prepared by spiking 5 × 10^3^ SK-BR-3 cells into 10^6^ Jurkat cells and was resuspended in 200 µL Dulbecco's phosphate-buffered saline (DPBS), which was used as the model sample for the evaluation of the capture efficiency of the Cell Reveal^TM^ system.

Blood samples collected from pregnant women were then used for the cbNIPD study. The fnRBCs which have distinct cell markers, such as the cluster of differentiation 71 (CD71), glycophorin A (GPA), the cluster of differentiation 36 (CD36), and epsilon hemoglobin, permitting to be isolated from the maternal blood [38,39,40,41] were chosen as the target for genetic analysis. The cluster of differentiation 45 (CD45) expressed on all white blood cells (WBCs) but not on fnRBCs was used as a negative selection marker for the fnRBC capture. Fourteen pregnant women with singleton pregnancies (at gestational age (GA) of 13^+4^–27^+5^ week^+day^ who have received invasive procedures (chorionic villus sampling or amniocentesis) and with confirmed fetuses that have evident non-maternal genomic markers, including 4 cases with de novo pathogenic copy number changes (9p24.2p23 deletion, *n* = 1; 10q25.2q26.12 deletion, *n =* 1; 21q22.11q22.3 deletion, *n* = 1; and 22q11.21 deletion, *n* = 1), 4 cases with trisomic chromosomes (48,XXY,+18, *n* = 1; 47,XY,+18, *n* = 2; and 47,XY,+21, *n* = 1), and 6 euploid cases with a male karyotype (46,XY, *n* = 6) were recruited in this study. For each case, approximately 8 mL of venous blood were collected and stored in the BD vacutainer ® with acid citrate dextrose (ACD) solution A (Becton, Dickinson and Company, Franklin Lakes, NJ, USA). This study was approved by the Ethics Committee of the Changhua Christian Hospital, Changhua, Taiwan (project ID: CCH-IRB-171215). All participants gave written informed consent before the study began.

### 2.2. Coral Chip Manufacture

**Figure 1:**
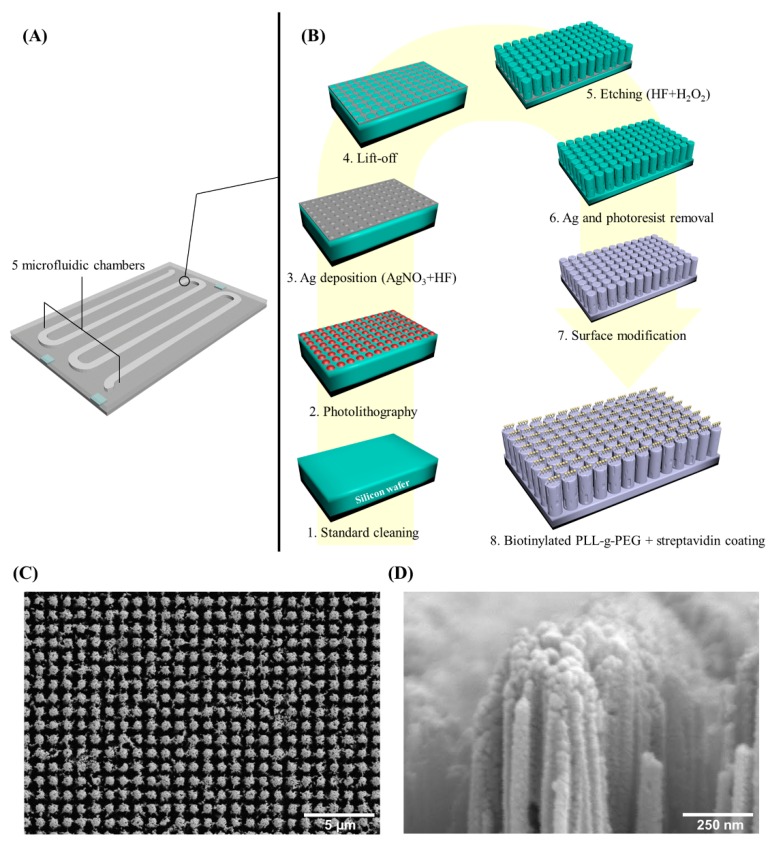
The silicon-based microfluidic Coral Chip. (**A**) An exemplified Coral Chip with 5 microfluidic chambers. (**B**) The manufacturing follow chart of the Coral Chip chamber surface: 1. standard cleaning, 2. photolithography, 3. Ag deposition, 4. liftoff, 5. etching, 6. Ag and photoresist removal, 7. surface modification, and 8. biotinylated PLL-g-PEG + streptavidin coating. (**C**) Top view and (**D**) lateral view of a scanning electron microscope (SEM) image of the Coral Chip.

The Coral Chip is a silicon (Si)-based chip with a porous morphology on the inside of microfluidic chambers that are capable to capture targeted cells from a cell mixture. The chip is fabricated using the metal-assisted chemical etching technology as previously described [19], with minor modifications (Figure 1). Briefly, 5 instead of 3 microfluidic chambers were created in this chip to extend the surface area for cell capture. Moreover, the fabrication sequence was revised. The starting materials of p-type (100) Si wafers followed the standard cleaning to remove the environmental contaminants. Then, the plasma-enhanced chemical vapor deposition deposited a SiNx layer for a hard mask on Si wafers. The chip’s pattern was defined by using the standard photolithography technique and the inductively coupled plasma etched SiNx hard mask pattern. A 20 nm Ag film was evaporated onto surfaces of wafers and was lift off the metal caps on the photoresist. The wafers were etched in a HF/H_2_O_2_ mixture solution, with a concentration of 4.8 M and 0.3 M, respectively. After finishing the Ag removal, the hard mask SiNx was etched in a 125 °C H_3_PO_4_ and a Si nanostructure with porous morphology was formed. The wafers were cut into a standard-sized Coral Chip to fit the microfluidic component of the Cell Reveal^TM^ system. The surface of the chip was finally modified by silane deposition and coated with biotinylated PLL-g-PEG + streptavidin.

When the chip was used for cell capture, the potential targeted cells are pre-labeled with biotin-conjugated monoclonal antibodies. The strong interaction between streptavidin and biotin enables a high efficiency for cell capture by the chip.

### 2.3. Cell Spiking Test

**Figure 2:**
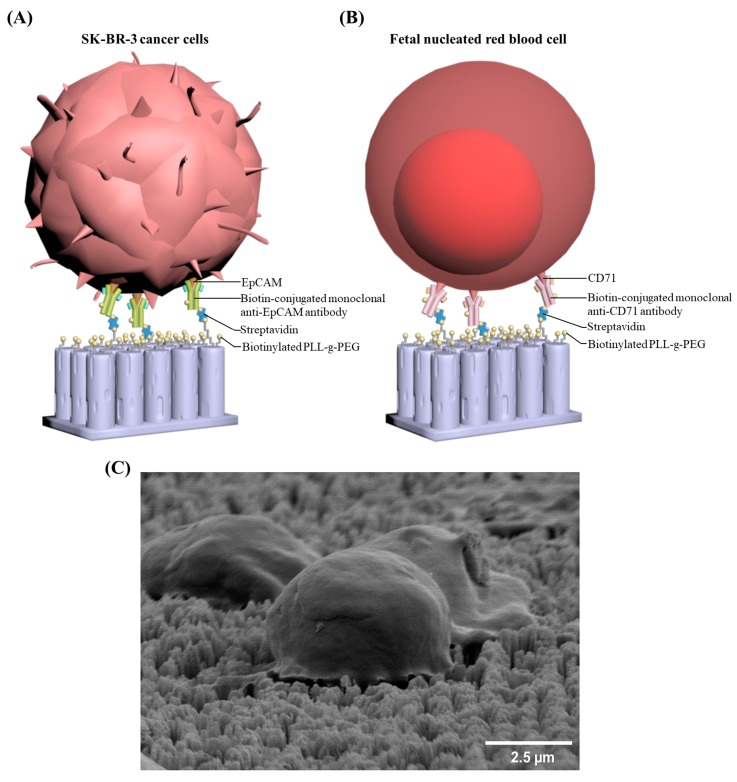
Rare cell captures by the Coral Chip: The Coral Chip surface is coated with biotinylated PLL-g-PEG + streptavidin, and the potential targeted cells are pre-labeled with biotinylated antibodies. The strong interaction between streptavidin and biotin enhances the capturing effect. (**A**) A schematic diagram of the SK-BR-3 cancer cells captured from an artificial cell mixture with a large amount of Jurkat cells as the background. (**B**) A schematic diagram of the fetal nucleated red blood cells (fnRBCs) captured from the peripheral blood of pregnant women. (**C**) Scanning electron microscope (SEM) micrographs of the targeted cells captured on Coral Chip.

The mixed cell suspension of SK-BR-3 and Jurkat cells was injected into the Cell Reveal^TM^ system for the evaluation of the capture efficiency. The subsequent procedures were automatically carried out by the system with a cell flow rate of 0.6 mL/h. The inputted cell suspensions were fixed in 4% paraformaldehyde. Then, Triton X-100 (0.1%) and 2% BSA (bovine serum albumin) were added to increase the cellular permeability and to prevent nonspecific binding sites. The antibody used for the primary capture of SK-BR-3 cells is anti-EpCAM (Figure 2A). Then, the captured cells were treated with anti-CK and anti-CD45 antibodies. Finally, fluorescence-labeled secondary antibodies were used to stain the targeted cells. The chips are examined using a fluorescence microscope equipped with a built-in automated inspection and image analysis system to filter out images of Jurkat cells for further analyses. The SK-BR-3 cells can therefore be targeted, identified, and enumerated. Image analyses with the count-in/filter-out criteria for SK-BR-3 and Jurkat cells are EpCAM+/CK+/CD45−/Hoechst+ and EpCAM-/CK-/CD45+/Hoechst+, respectively. Data for the test were repeated in quadruplicate. The capture efficiency and false capture rate were determined as the number of captured SK-BR-3 cells divided by the total number of spiked SK-BR-3 cells and the number of captured Jurkat cells divided by the total number of background Jurkat cells, respectively.

### 2.4. Fetal Nucleated Red Blood Cells (fnRBCs) Capture

The whole blood sample was flown through the automated Cell Reveal^TM^ system (with a rate of 0.6 mL/h) and then fnRBCs were captured by Coral Chip. For each test run, 4 Coral Chips can be used simultaneously to analyze 8 mL blood (2 mL blood per chip). The antibody used for primary capture of fnRBCs is anti-CD71 (Figure 2B). The captured cells were then treated with anti-GPA and anti-CD45 antibodies and stained by fluorescence-labeled secondary antibodies. As a result, the fnRBCs can be automatically targeted, identified, and enumerated by image analyses with the count-in/filter-out criteria CD71+/GPA+/CD45−/Hoechst+ for fnRBCs and CD71-/GPA-/CD45+/Hoechst+ for maternal WBCs. 

### 2.5. Fluorescence in Situ Hybridization (FISH)

FISH was performed directly on Coral Chip capturing for fnRBCs. Prior to hybridization, the formaldehyde on Coral Chip were treated by a 10 mM sodium citrate at 90 °C for 20 min; followed by being immersed in 0.1% Triton-X at room temperature for 10 min; and then followed by serial washes of 0.2 N HCl at 25 °C for 20 min, double distilled water at 25 °C for 3 min, 2× saline-sodium citrate (SSC) at 25 °C for 3 min, and an immersion of Vysis pretreatment solution (1 N NaSCN) (Abbott, Lake Bluff, IL, USA) at 25 °C overnight. Then, the Coral Chip was deposited in purified water at 25 °C for 1 min, 2× SSC at 25 °C for 5 min (repeated two times), pepsin solution (10 μL 10% Pepsin/40 ml 0.01 N HCl) at 37 °C for 3 min, and 2× SSC at 25 °C for 5 min (repeated two times). Finally, the Coral Chip was immersed in 70% ethanol at 4 °C for 1 min, 85% ethanol at 4 °C for 1 min, and 100% ethanol at 4 °C for 1 min and dried at 50 °C for 5 min. The interphase FISH for chromosomes 18, 21, or Y was conducted on captured fnRBCs. For the hybridization experiment, the Coral Chips were dehydrated in an ethanol series and hybridized overnight in a moist chamber at 37 °C. The chips were washed for 2 min in 0.4× SSC at 70 °C and for 5 min in 4× SSC, 0.1% Tween 20 at room temperature and blocked in 4× SSC, 3% bovine serum albumin (BSA), 0.1% Tween 20 at 37 °C for 30 min. The hybridization signal was detected with a Nikon-Ni-E microscope system (Nikon, Tokyo, Japan). The chromosomes were counterstained with 0.125 μg/mL 4′,6-diamidino-2-phenylindole (DAPI) in Antifade (Vysis, Downers Grove, IL, USA). The FISH analyses were performed using the Aquarius® FAST FISH Prenatal kit (Cytocell, Cambridge, UK) for trisomy 18 and 21 fetuses (the chromosome 18 probe for the centromere of chromosome 18 (D18Z1) and the chromosome 21 probe for D21S270, D21S1867, D21S337, D21S1425, and D21S1444 were labeled with aqua and orange fluorophores, respectively) and using the centromeric enumeration probe (CEP) X SpectrumOrange/Y SpectrumGreen DNA probe kit (Vysis, Downers Grove, IL, USA) for euploid male fetuses (the chromosome X probe for Xp11.1q11.1 alpha satellite DNA and the chromosome Y probe for Yq12 satellite III were labeled with orange and green fluorophores, respectively). 

### 2.6. Captured Cells Recovery

**Figure 3:**
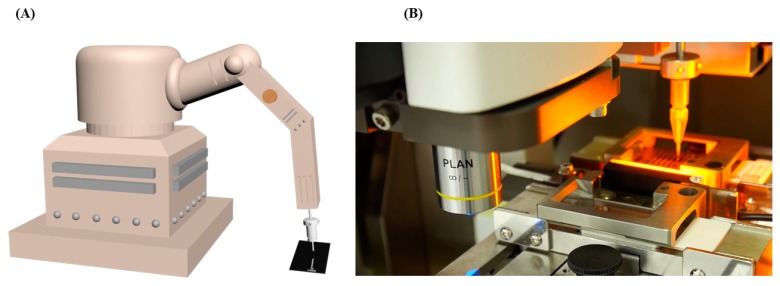
An automated cell picker. (**A**) A schematic diagram of the cell picker machine. (**B**) The targeted cell enriched on the Coral Chip can be automatically recovered by the cell picker.

The cells captured on Coral Chip (i.e., SK-BR-3 cells and fnRBCs) (Figure 2C) can be recovered by an automated cell picker which is upgraded from the manual micropipetting system that we previously reported [19]. That is, the target cell location coupled with the coordinates were acquired by the Cell Reveal^TM^ system. Then, the Cell Reveal^TM^ system removed a computer lid covering the Coral Chip during the cell capture process and exposed the microfluidic chamber to the cell picker. Finally, the in-house developed software coordinates the fluorescent microscope and the pipetting system to recover the target cells (Figure 3).

### 2.7. Whole Genome Amplification (WGA)

Five to 15 captured cells recovered from the Coral Chip were pooled in a single 0.2 mL PCR tube. The recovered cells were subjected to whole genome amplification (WGA) using the PicoPLEX Single Cell WGA Kit (Takara Bio, Mountain View, CA, USA) following the manufacturer’s instructions. Amplified DNA was purified using the QIAquick PCR purification kit (Qiagen, Hilden, Germany). The DNA purities and concentrations were examined by a Nanodrop 2000 spectrophotometer (Thermo Fisher Scientific, DE, USA).

### 2.8. Array Comparative Genomic Hybridization (aCGH)

Approximately 1 μg of purified WGA DNA was fluorescently labeled with Cy3 d-CTP or Cy5-dCTP using the SureTag DNA Labeling Kit (Agilent Technologies, CA, USA) and then cleaned up by a Microcon YM-30 centrifugal filter unit (Millipore, MA, USA). The yield DNA was hybridized with a CytoScan 60 × 8K microarray chip (Agilent customer array, Changhua Christian Hospital, Changhua, Taiwan) following the manufacturer’s instructions. The image on a chip was acquired with a G4900DA SureScan microarray scanner (Agilent Technologies) and analyzed with Agilent Genomic Workbench software (Agilent Technologies) for chromosome gain or loss across all 24 chromosomes. Aberrations were detected using the default setting with the z-score algorithm conjugated with a filter of a minimum of 5 Mb aberrations.

### 2.9. Next Generation Sequencing (NGS)

Approximately 1 μg of purified WGA DNA was used for library construction by the Ion Xpress Plus gDNA Fragment Library Preparation Kit Set (Life technologies, Carlsbad, CA, USA) following the manufacturer’s instructions. The quantity of library was determined using Qubit dsDNA HS assay kits (Life technologies) with Qubit fluorometers (Life technologies). The template-positive Ion Sphere Particles were generated using Ion PGM Hi-Q Template Kits (Life technologies) with the Ion OneTouch 2 Instrument (Life technologies) and then enriched with the Ion OneTouch ES Instrument (Life technologies). Sequencing was performed on the Ion Torrent PGM Instrument (Life technologies) platform with the Ion PGM Hi-Q Sequencing Kit and Ion 316 chip (Life technologies). The sequencing data analysis was performed by using the cloud-based the Ion Reporter^TM^ Server System (https://ionreporter.thermofisher.com/ir/).

### 2.10. Short Tandem Repeat (STR) Analysis

The STR analysis was performed for gender determination in order to confirm that the circulating cells captured are indeed from male fetuses instead of maternal origin. The GenomeLab™ Human STR Primer Set kit (Beckman Coulter, Brea, CA, USA) containing the primer pair of gender-specific AMEL locus was used to analyze the STR pattern on the GenomeLab™ GeXP Genetic Analysis System (Beckman Coulter). The data were then analyzed by the FRAGMENTS application program (Beckman Coulter).

## 3. Results

### 3.1. Capture Efficiency Estimated by Cell Spiking Test

Four model samples, each prepared by spiking SK-BR-3 cells into background Jurkat cells, were used to evaluate the capture efficiency of the Cell Reveal^TM^ system. The cell capture experiment was carried out according to the procedure mentioned above (Section Cell Spiking Test). The mean of the capture rate is 88.17% (range: 80.24%–94.56%). The mean of the false capture rate is close to 0% (range: 0%–0.0007%) (Table 1).

### 3.2. Circulating fnRBC Captured by Coral Chip

**Figure 4:**
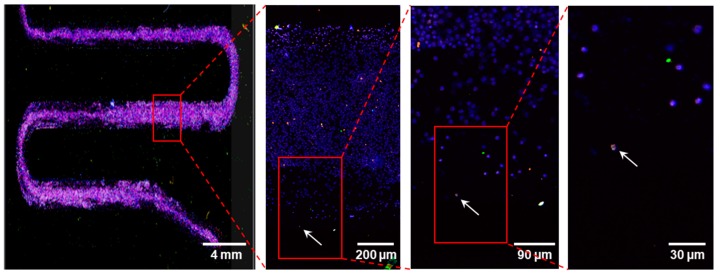
Images of fetal nucleated red blood cells (fnRBCs) enrichment on chambers of a Coral Chip. An fnRBC identified by immunocytochemistry is indicated by an arrow.

**Figure 5:**
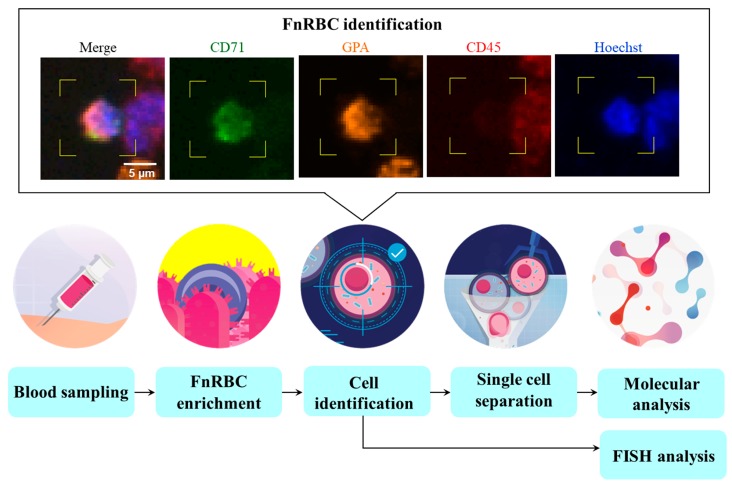
The process flow diagram of a cell-based noninvasive prenatal diagnosis (cbNIPD) by the fetal nucleated red blood cells (fnRBCs) enrichment strategy. The fnRBCs were identified based on the count-in/filter-out criteria of CD71+/GPA+/CD45−/Hoechst+.

In every 2 mL of the maternal blood being tested on 1 Coral Chip, the circulating fnRBCs were always captured in all the 14 pregnant women examined (Table 2). The fnRBCs were enriched on the chip (Figure 4) and identified based on the count-in/filter-out criteria of CD71+/GPA+/CD45−/Hoechst+ by a fluorescence microscope equipped with a built-in automated inspection and image analysis system (Figure 5). The cells automatically captured by system were rechecked manually. All the captured cells passed the count-in/filter-out criteria of fnRBCs, suggesting a low false capture rate. The number of captured fnRBCs were 2–71 cells per 2 mL of maternal blood. The total numbers of fnRBCs captured were 273 cells. As a result, the overall capture rate is estimated as 9.75 fnRBCs per ml maternal blood per individual (Table 2).

### 3.3. FISH

**Figure 6:**
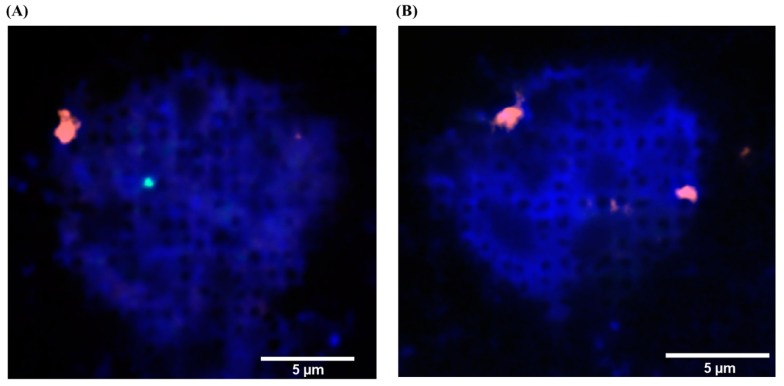
The fluorescent in situ hybridization (FISH) for cells on a Coral Chip. The cells are from the blood of a pregnant women with an euploid male fetus (case 13 in Table 2). The FISH was directly performed on the chip using the CEP X SpectrumOrange/Y SpectrumGreen DNA probe kit (Vysis, Downers Grove, IL, USA). (**A**) The fetal nucleated red blood cell (fnRBC) can be distinguished from (**B**) the maternal white blood cell (WBC) by the signals of chromosome X and Y: the fnRBC has 1 orange and 1 green signal, and the maternal WBC has 2 orange signals.

Interphase FISH for the captured fnRBCs from the blood of the 4 pregnant women with a fetus of trisomy 18 or trisomy 21 (cases 5–8 in Table 2) and for 5 of the 6 pregnant women with euploid male fetuses (cases 9–13 in Table 2) revealed correct diagnoses in all cases. For each case, at least 2 fnRBCs were examined on the chip. Figure 6 exemplified a FISH result using the CEP X SpectrumOrange/Y SpectrumGreen DNA probe kit (Vysis, Downers Grove, IL, USA) for a pregnant women with a euploid male fetus (case 13 in Table 2). The fnRBC can be distinguished from the maternal WBC by the signals of chromosome X and Y: the fnRBC has 1 orange and 1 green signal, and the maternal WBC has 2 orange signals (Figure 6).

### 3.4. Captured Cells Recovery

The recovery rate for the targeted cells is estimated to be 90%. About 10% of cells were lost when they were pulled out from the chip by the micropipetting system.

### 3.5. WGA

All pooled captured cells underwent WGA successfully except those with a total number of cells that were too few (namely, less than 5 cells) to reach the amplified threshold for subsequent molecular genetic analyses by aCGH, NGS, or STR analyses. Overall, the SK-BR-3 cell WGA DNA as well as the fnRBC WGA DNA from 11 prenatal cases (cases 1–6, 8, 11–14 in Table 2) were obtained. The WGA products were 30 uL in total, with a concentration ranged from 150–275 ng/uL.

### 3.6. aCGH and NGS Analysis

**Figure 7:**
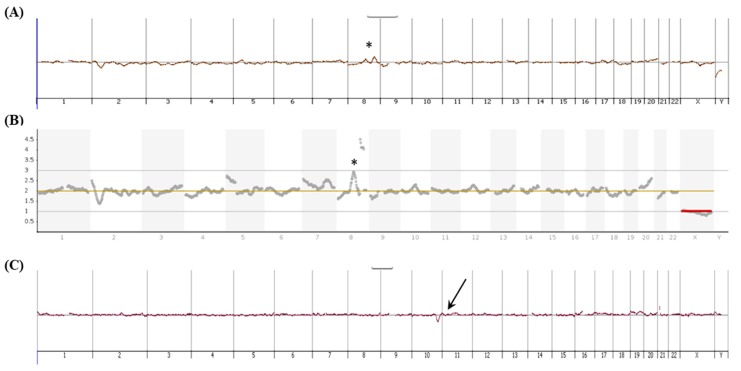
The molecular analyses for targeted cells enriched on and then captured from the Coral Chip. (**A**) The array comparative genomic hybridization (aCGH) and (**B**) the next generation sequencing (NGS) for the SK-BR-3 cancer cells. The recognizable genomic imbalance [42] in chromosome 8 was denoted by a star. (**C**) The aCGH for the circulating fetal cells with a de novo deletion in chromosome 10q25.2q26.12 (i.e., the case 2 in Table 2). The 10q25.2q26.12 deletion is indicated by an arrow. The DNA used for the molecular analyses was extracted from 4–5 captured cells and then amplified by the PicoPLEX Single Cell WGA Kit (Takara Bio, Mountain View, CA, USA).

For SK-BR-3 cells, both aCGH and NGS analyses were performed, and the recognizable genomic features of the SK-BR-3 cell line [42] were identified (Figure 7A,B). For the four prenatal cases with de novo pathogenic copy number changes (cases 1–4 in Table 2), aCGH were performed and the results are consistent with the fetal genetic features pre-acquired by aCGH of amniotic fluid. An exemplified aCGH result for the captured fnRBCs (case 2 in Table 2) is showed in Figure 7C.

### 3.7. STR Analysis

An STR analysis was performed in fnRBCs of 1 prenatal euploid male case (case 14 in Table 2) for gender determination. The results demonstrated the captured fnRBCs contain the informative STR marker on chromosome Y and are indeed of fetal origin (data not shown).

## 4. Discussion

Although a number of research groups had made tremendous efforts on isolating CFCs (especially fnRBC) and tried to apply the technology to clinical utility, very few had actually reported successful results [19,21,22,23,24]. Meanwhile, some of the published studies have the potential to become a laboratory developed test, but the laborious experimental steps made those published reports questionable if these tests can truly turn into a reliable and stable system being adopted by clinical cytogenetics laboratories. Our cell capture system is nearly automated in both processes of the cell capture and recovery. Moreover, the Coral Chip used is manufactured by photolithography and etching, a process easy to achieve standardization and production compared with other nanostructure wet etching methods. As a result, this system has the scalability and potential to become an in vitro diagnostic which may change the landscape now that it has been dramatically reshaped by the popular cffDNA testing. Currently, most of the emerging platforms are targeted at trophoblasts [16,28,31]. It is reasonable since trophoblasts are much larger than the background of maternal WBCs and adds another useful determinant to differentiate trophoblasts from maternal cells, and having an intact trophoblast can still provide much more information than the fragmented cffDNA degraded from trophoblasts. However, fnRBC is indeed representative of the true fetal genome, and therefore, its priority of cbNIPD should be higher than trophoblasts. In our previous proof-of-principle pilot [19], we demonstrated the feasibility of our platform to detect fetal aneuploidy by using common trisomies (i.e., trisomy 13, 18, and 21). Here, we further expand our case series into those with de novo chromosome copy number changes and those carrying male fetuses, and we demonstrated the cells being captured are indeed of fetal origin by different genetic analyses including FISH, aCGH, and STR.

It is now well-known the trend of the variation of the fetal DNA fractions during the whole gestation, as well as the possible confounding factors (e.g., material body mass index, fetoplacental mosaicism, anticoagulation therapy, vanishing twin syndrome, and genetic chimerism caused by blood transfusion or maternal malignancy) that may cause false-positives or false-negatives by the cffDNA testing [7,15,43]. On the contrary, a recent report showed that the maternal body mass index (BMI) has no effect on the number of CFCs being captured [28], a fact hinting that cbNIPD may have much fewer limitations than cffDNA testing and a greater potential to achieve a true NIPD in the future. However, it should be highlighted that any cbNIPD platforms must be able to demonstrate its feasibility through prospective, double-blinded, large-scale clinical trials to convince the clinical communities, hopefully in the near future, that indeed it is a workable solution and can then compete with the now very successful cffDNA NIPT. It can also be anticipated that new confounding factors may affect the accuracy of cbNIPD theoretically, such as fetomaternal hemorrhage, a not uncommon complication during the gestation [44].

## 5. Conclusions

Our results demonstrated that the Cell Reveal^TM^ system has a high capture efficiency and can be used for fnRBC capture and recovery that is feasible for the genetic diagnosis of fetuses without invasive procedures. However, to convince its clinical utility in cbNIPD, a prospective, large-scale, randomized study is needed.

## Figures and Tables

**Table 1:** A summary of the cell spiking test: In each sample, 5 × 10^3^ SK-BR-3 breast cancer cells were mixed with 10^6^ Jurkat cells in 200 µL Dulbecco's phosphate-buffered saline (DPBS) and subjected to Cell Reveal^TM^ system to examine the capture efficiency.

Sample No.	No. of Captured SK-BR-3 cells	No. of Falsely Captured Jurkat Cells	Capture Rate for SK-BR-3 Cells (%)	False Capture Rate for Jurkat Cells (%)
1	4012	7	80.24	0.0007
2	4241	0	84.82	0
3	4683	0	93.06	0
4	4728	1	94.56	0.0001
Mean	4405	2	88.17	0.0002

**Table 2:** The validation of the cell-based noninvasive prenatal diagnosis (cbNIPD) in 14 pregnant women.

Case No.	MA (Year)	GA (Week^+day^)	Pre-acquired Fetal Genetic Condition	cbNIPD		
				No. of fnRBCs Captured (in 2 mL Maternal Blood)	Non-maternal Genomic Markers Used to Confirm the Fetal Origin of Captured Cells	Validated * Method
1	30	27^+5^	arr[GRCh37] 9p24.2p23 (2267812_13374304) × 1 dn	10	1. 9p24.2p23 deletion 2. Chr Y	aCGH [pooled 8]
2	38	20^+6^	arr[GRCh37] 10q25.2q26.12 (114393625_121720948) × 1 dn	47	1. 10q25.2q26.12 deletion 2. Chr Y	aCGH [pooled 13]
3	31	25	arr[GRCh37] 21q22.11q22.3 (35703384_48056450) × 1 dn	47	1. 21q22.11q22.3 deletion 2. Chr Y	aCGH [pooled 15]
4	40	18	arr[GRCh37] 22q11.21 (18894835_21505417) × 1 dn	18	22q11.21 deletion	aCGH [pooled 10]
5	28	15^+6^	48,XXY,+18	7	T18	FISH [4]
6	37	13^+4^	47,XY,+18	25	T18	FISH [10]
7	29	16	47,XY,+18	3	T18	FISH [3]
8	34	20^+6^	47,XY,+21	14	T21	FISH [6]
9	43	25^+6^	46,XY	3	Chr Y	FISH [3]
10	32	19	46,XY	2	Chr Y	FISH [2]
11	29	24^+6^	46,XY	10	Chr Y	FISH [6]
12	37	15	46,XY	10	Chr Y	FISH [4]
13	28	24	46,XY	71	Chr Y	FISH [22]
14	42	24	46,XY	6	Chr Y	STR analysis [pooled 5]

* The number in the bracket indicates the number or pooled number of captured cells used for validation. MA, maternal age; GA, gestational age; fnRBC, fetal nucleated red blood cell; Chr, chromosome; T18, trisomy 18; T21, trisomy 21; aCGH, array-based comparative genomic hybridization; FISH, fluorescence in situ hybridization; and STR, short tandem repeat.
